# Transcriptome analyses of a Chinese hazelnut species *Corylus mandshurica*

**DOI:** 10.1186/1471-2229-13-152

**Published:** 2013-10-05

**Authors:** Hui Ma, Zhiqiang Lu, Bingbing Liu, Qiang Qiu, Jianquan Liu

**Affiliations:** 1 Molecular Ecology Institute, Key Laboratory of Arid and Grassland Ecology, Lanzhou University, Lanzhou 730000, China

**Keywords:** Corylus mandshurica, Transcriptome, Adaptation, Divergence, Fungi/fungus, Cold/frigid, Taxol/paclitaxel

## Abstract

**Background:**

*Corylus* was renowned for its production of hazelnut and taxol. To understand the local adaptation of Chinese species and speed up breeding efforts in China, we analyzed the leaf transcriptome of *Corylus mandshurica*, which had a high tolerance to fungal infections and cold.

**Results:**

A total of 12,255,030 clean pair-end reads were generated and then assembled into 37,846 Expressed Sequence Tag (EST) sequences. During functional annotation, 26,565 ESTs were annotated with Gene Ontology (GO) terms using Blast2go and 11,056 ESTs were grouped into the Kyoto Encyclopedia of Genes and Genomes (KEGG) pathways using KEGG Automatic Annotation Server (KAAS). We identified 45 ESTs that were homologous to enzymes and transcription factors responsible for taxol synthesis. The most differentiated orthologs between *C. mandshurica* and a European congener, *C. avellana,* were enriched in stress tolerance to fungal resistance and cold.

**Conclusions:**

In this study, we detected a set of genes related to taxol synthesis in a taxol-producing angiosperm species for the first time and found a close relationship between most differentiated genes and different adaptations to fungal infection and cold in *C. mandshurica* and *C. avellana*. These findings provided tools to improve our understanding of local adaptation, genetic breeding and taxol production in hazelnut.

## Background

*Corylus* is an important genus, both economically and ecologically, in China. There is currently more than 4 million acres of natural hazel groves in northeastern China alone [1]. Its nuts, rich in unsaturated fat and vitamins, are widely consumed. Its leaves are used by local farmers to feed domestic silkworm [2]. Its stocks have been successfully used for grafting *Castanea henryi* to improve chestnut production and quality [3] and have also been shown to be an ideal substitute for logs of *Carpinus cordata* in *Ganoderma* culture [4]. A part from its clear economic importance, *Corylus* plays an important role in soil and water conservation owing to its strong root system and contributes to the sustainability of forests in this region [2].

*Corylus* species are also important sources of taxol (also named as Paclitaxel), which is an effective yet relatively expensive medicine for treatment of breast, ovarian and lung cancer [5-7]. Taxol was originally isolated from the bark of Pacific yew [8] and then later found to be present in the yew genus *Taxus*[9]. It was initially believed to occur only in gymnosperms, but was recently identified in leaves and fruits of a hazelnut species (*C. avellana*) [10]. Further studies validated this finding by showing that *in vitro* hazel cell cultures produce taxol and taxanes, indicating that it is not exclusively produced by symbiotic fungi [11-13]. Taxol was recently discovered in another hazelnut species, *C. mandshurica* (synonym to *C. sieboldiana*) [14]. However, except for the *Corylus* species as well as a few other species like *Magujreothamnus speciosus*, *Morinda citrifolia*, *Justicia gendarussa* and *Yunnanopilia longistaminata*[15,16], few angiosperm species have been reported to contain taxol or its derivatives. Interests in taxol production from hazel trees, especially from its leaves, have grown rapidly with the aim of conserving endangered yew species [17].

*C. mandshurica* is widely distributed in northeastern China and its nuts are characterized by a thin husk and high shelling percentage [18]. The nuts from this species are of higher quality in flavor and taste, and therefore command a higher price than the nuts from *C. avellana*. Moreover, *C. mandshurica* is highly resistant to Eastern Filbert Blight [19], a fungus that causes seriously damage to most commercially grown cultivars of *C. avellana* in the US [20], and has exceptional cold resistance; it is able to survive a frigid winter of -48°C [21,22]. All these traits make it a very desirable target for developing improved selections and breeding material [18,23]. Interspecific crossing and breeding experiments have been attempted between *C. mandshurica* and the commercial species *C. avellana*[1,22-26]. Molecular breeding aided by microsatellite marking has also been reported [27,28].

Next generation sequencing is a quick and cost-effective method for surveying the complete coding sequence of a genome. Much progress has been made in obtaining longer sequence reads, and many tools and algorithms have been developed to allow assembly of short reads. Despite the ever-increasing sequencing data, the Expressed Sequence Tags (ESTs) from *C. avellana* have only recently been released [29] and remain the only available large-scale sequencing data for the *Corylus* genus. In this study, we sequenced the leaf transcriptome of *C. mandshurica* native to China. Our aims were (1) to explore how homologous genes of two hazel species have differentiated to give their contrasting adaptations, and (2) to identify the possible genetic basis of taxol production in the genus *Corylus* by transcriptome assembly and gene annotation.

## Results and discussion

### Sequencing and assembly

**Figure 1:**
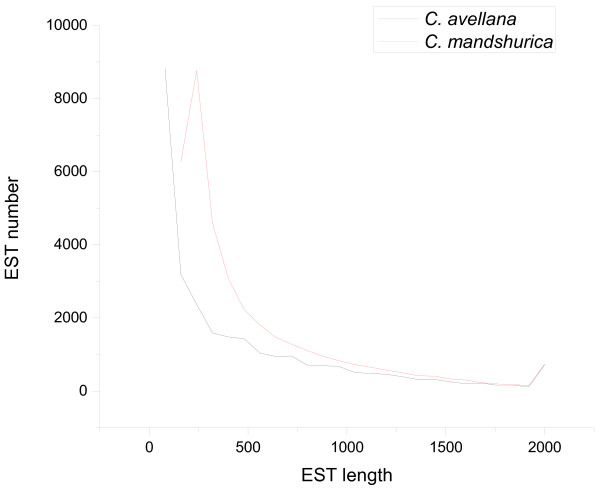
**The length distribution of assembled ESTs from transcriptomes of *C. mandshurica *and *C. avellana*.** ESTs are counted at an interval of 80 bp, with ESTs longer than 2000 bp counted as 2000 bp. It is clear that more ESTs are assembled in the transcriptome of *C. mandshurica* than *C. avellana* at length intervals after 160 bp. (Trinity has a minimum length of 200 bp in transcriptome assembly).

After strict quality control, 12,255,030 clean pair-end reads were assembled into 37, 846 ESTs longer than 200 bp using Trinity [30]. The contig N50 was 799 bp and 8,328 ESTs had longer sequences. A total of 37,652 coding DNA sequences (CDSs) were predicted to have an average length of 431 bp using Orfpredictor [31]. Comparison of our assembly with the Jefferson transcriptome assembly on *C. avellana* was shown in Table 1. It could be seen that the contig N50 of our assembly was slightly lower, which was partly due to the large increase in the number of assembled sequences. It was apparent from EST length distribution (Figure 1) that our assembly had more sequences at all length intervals beyond 160 bp. The assembled EST sequences of C. mandshurica in fasta format were available in Additional file 1.

**Table 1:** **Comparison of transcriptome assembly and coding sequence prediction for *Corylus mandshurica *and *Corylus avellana***

	**EST**		**CDS**	
	***C. mandshurica***	***C. avellana***	***C. mandshurica***	***C. avellana***
Average Length	580	532	431	377
Length Range	201 ~ 6821	80 ~ 5490	30 ~ 4890	42 ~ 4143
Numbers	37846	28255	37652	28167
N50 Length	799	961	594	651
Sequences (longer than N50)	8328	4991	8028	4945

**Figure 2:**
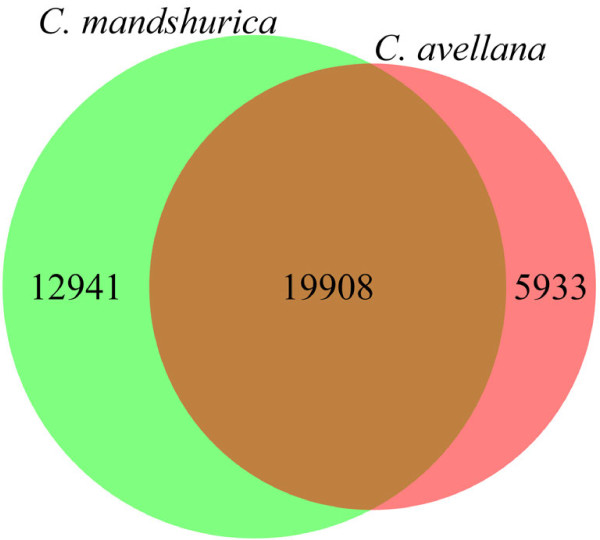
**The contigs from *Betula nana *genome mapped by transcriptomes of *C. mandshurica *and *C. avellana*.** The contigs mapped uniquely by ESTs from *C. mandshurica* may represent novel genes specifically expressed in it or different fragments of the same genes unidentified in the transcriptome of *C. avellana*.

Given the recently released genome of *Betula nana*[32], we used BLAT [33] to map our transcriptome assembly against this genome that currently consisted of 551,915 contigs. We found that 32,078 ESTs mapped to 32,849 contigs. In comparison, 25,073 ESTs out of the Jefferson transcriptome assembly mapped to 25,841 contigs, with 19,908 contigs shared between the two hazelnut transcriptome assemblies (Figure 2). The ESTs that mapped to unique contigs might represent different genes specifically expressed in each species or different fragments of the same genes due to the fragmentary nature of the current *Betula* genome and the limited sequencing depth of the transcriptomes. Thus, our transcriptome analysis revealed many novel EST sequences for the *Corylus* genus that could not be identified from the Jefferson transcriptome assembly and helped locate the genomic locus for each EST, which had important implications for the development of further breeding markers of the *Corylus* species.

### Functional annotation

**Figure 3:**
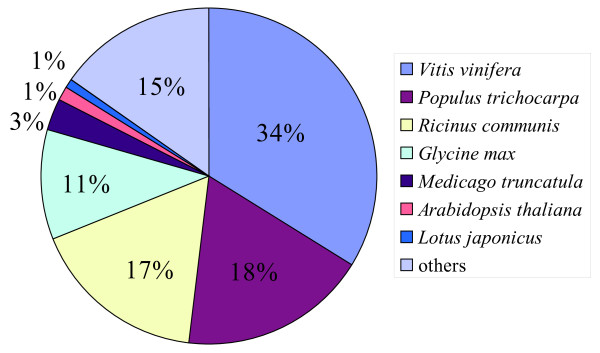
The percentage of top blast hits by species.

To functionally classify the assembled ESTs, a homology-based approach was adopted in transcriptome annotation. A total of 30,536 ESTs gave hits on performing BLASTX searches [34] in the NCBI non-redundant protein database using an E-value cutoff of 1e-5, accounting for 80.7% of all assembled sequences. When sorting the top blast hits by species, *Vitis vinifera* was ranked first with 10,321 top blast hits, followed by *Populus trichocarpa* and *Ricinus communis* with 5,537 and 5,155 top blast hits, respectively (Figure 3). In addition, 26,565 ESTs were annotated with Gene Ontology (GO) terms using Blast2go [35] and 11,056 ESTs were annotated into Kyoto Encyclopedia of Genes and Genomes (KEGG) pathways with KEGG Automatic Annotation Server (KAAS) [36] using the Single-directional Best Hit (SBH) method.

### Identification of highly differentiated genes in the transcriptomes of *C. mandshurica* and *C. avellana*

**Figure 4:**
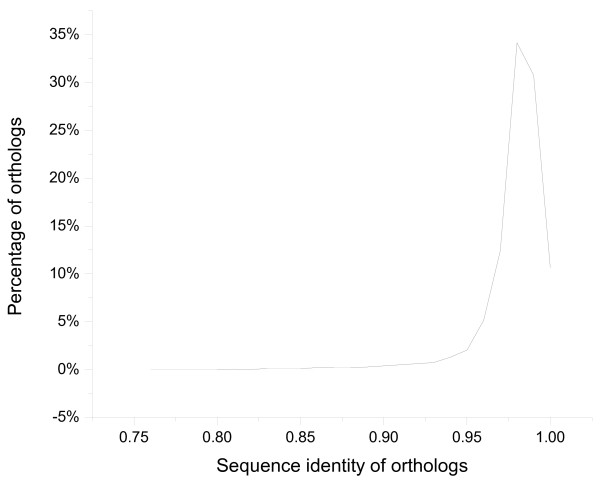
**The distribution of sequence identity in blast regions for all orthologs.** 98.7% orthologs have sequence identity no less than 90%; 87.9% orthologs have sequence identity no less than 97%. To exclude distantly homologous sequences, orthologs with sequence identity less than 90% are discarded in GO enrichment analysis.

Since *C. mandshurica* and *C. avellana* were closely related species but with contrasting adaptations, our first goal was to identify which genes were highly differentiated. Using the available EST sequences for these two species, we performed a reciprocal blast to obtain best hit orthologs and compared both the sequence identities and presence of **In**sertion/**Del**etion (INDEL) according to BLASTN outputs. Since 98.7% of orthologs showed a sequence identity higher than 90% (Figure 4), we set a sequence identity of 90% as the low threshold in ortholog validation to exclude the presence of distantly related homologs. Because we were interested in orthologs with relatively great divergence between *C. mandshurica* and *C. avellana*, we took orthologs with the low sequence identity (less than 97%), which account for 10.4% of all orthologs, as the highly differentiated genes. Furthermore, INDEL might cause different reading frames in coding regions of the two sets of orthologous ESTs [37]. It might also cause mRNA secondary structure change [38] in the coding and noncoding regions with alternative roles in transcriptional polyadenylation site selection [39], pre-mRNA splicing [40], mRNA stability, translation efficiency and protein folding [41,42]. Therefore, INDEL was also used as an indicator for sequence divergence. Thus, orthologs with gaps in the BLAST alignment were taken as another set of highly differentiated genes. Next, we performed separate GO enrichment analyses on these two types of differentiated orthologs using the WEGO web server [43]. GO terms for all orthologs and the two types of highly differentiated orthologs were available in Additional files 2, 3, 4).

**Figure 5:**
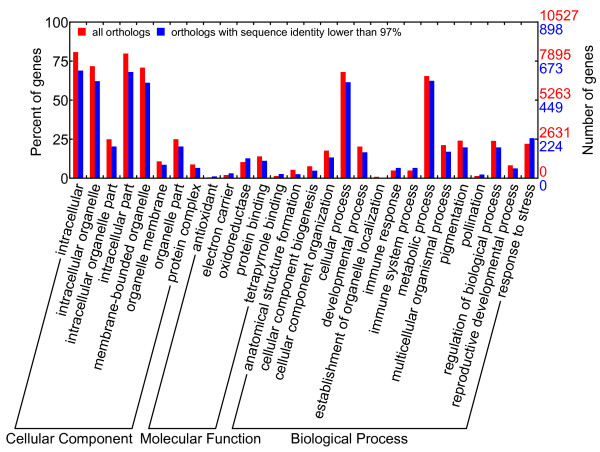
**GO enrichment on orthologs with sequence identity lower than 97%.** Direct parent GO terms in biological process domain are displayed for simplicity. The GO terms in the biological process domain that show increase in ESTs with lower sequence identity are related to immune response and response to stress.

**Figure 6:**
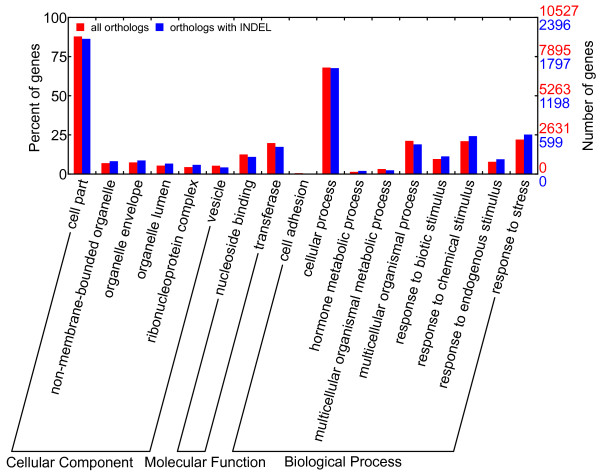
**GO enrichment on orthologs with INDEL.** Direct parent GO terms in biological process domain are displayed for simplicity. The GO terms in the biological process domain that show increase in ESTs with INDEL are related to hormone metabolic process and response to various stimuli (including response to stress).

**Figure 7:**
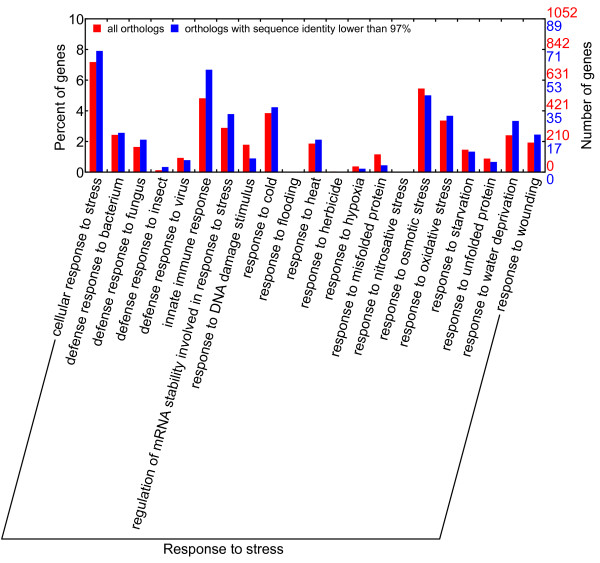
**GO terms under response to stress in GO enrichment on orthologs with low sequence identity.** GO terms related to defense against bacteria and fungi (including innate immune response), tolerance to cold and heat, and salt/drought/water stress show increase in ESTs with lower sequence identity.

**Figure 8:**
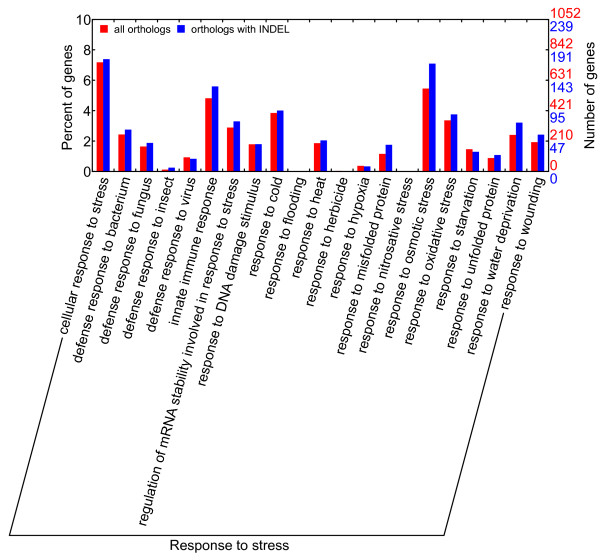
**GO terms under response to stress in GO enrichment on orthologs with INDEL.** GO terms related to defense against bacteria and fungi (including innate immune response), tolerance to cold and heat, and salt/drought/water stress show increase in ESTs with INDEL.

According to the GO enrichment analyses (Figures 5 and 6), orthologs from most statistically significant GO terms were conserved in the respective sequences as they generally contained a low percentage of orthologs with a sequence identity lower than 97% or orthologs with INDEL. The conserved GO categories comprised GO terms in cellular process, developmental process and metabolic process (except hormone metabolic process) in the biological process domain for both species. All these processes were essential for plant survival. The divergent GO categories comprised immune, pollination and response to stress in the biological process domain for the GO enrichment of orthologs with sequence identities lower than 97%. The orthologs with INDEL were enriched in hormone-related or various stimuli-related GO terms in the biological process domain (including hormone metabolic process and responses to various stimuli, especially response to stress). These findings suggested that *C. mandshurica* and *C. avellana* had become genetically differentiated whilst adapting to their different habitats. Stress response genes were more prone to both sequence substitution and insertion/deletion, with occurrences of 25.6% and 25.2% among all differentiated ESTs, respectively. A close examination of GO terms under response to stress (Figures 7 and 8) revealed that three main categories displayed increased sequence divergence, including genes participated in defenses to bacteria and fungi, genes involved in cold tolerance and genes related to salt/drought/water stress. As *C. mandshurica* has better adapted to fungal infection and cold stress than *C. avellana*, further study of the highly divergent genes in *C. mandshurica* could identify the key genes responsible for the resistance to fungal infection and cold pressure. However, because orthologs were not necessarily one to one match between two species when gene duplication occurred after speciation, the identified orthologs could be either true alleles or different copies of the same family in the genomes. Under the latter scenario, differential expressions of the genes at both time and space should be carefully examined, which might also represent one of the adaptation mechanisms in this species.

### Genes responsible for taxol synthesis

According to KEGG annotation, 29 ESTs were found to be involved in the terpene synthesis pathway. These included genes involved in isopentenyl-PP (IPP) synthesis in both the mevalonate and MEP/DOXP pathways and genes responsible for geranyl-PP and geranyl-geranyl-PP (GGPP) synthesis. The committing step for taxol production was the conversion of GGPP to taxa-4(5)-11(12)-diene in the diterpenoid biosynthesis pathway; however, genes involved in this reaction, as well as the following processes, were absent from our KEGG annotation. This was also encountered in the KEGG annotation of *C. avellana* transcriptome. Nonetheless, 31 ESTs (Table 2) were found to be homologous to the prototype genes participating in taxol synthesis in yew species, with sequence identities ranging from 23.93% to 50.32%. This was similar to the sequence identities of 40% ~ 44.1% reported in some taxol-producing fungi [44] and was close to the maximal sequence identity of around 40% ~ 49.3% found between these genes and the available proteins from other plant species in the NCBI non redundant protein database (Table 3). In addition, 6 ESTs were found to be homologous to WRKY, and 8 ESTs homologous to JAMYC. These two transcriptional factors had been reported to induce taxol synthesis [45,46].

**Table 2:** **ESTs homologous to genes involved in taxol synthesis in *Taxus***

**EST ID**	**Hit Protein GI**	**Identity (%)**	**Length**	**Description**
comp37211_c1_seq1	386304248	36.51	189	10-deacetylbaccatin III-10-O-acetyl transferase, partial
comp42386_c0_seq1	28558088	28.85	104	3′-N-debenzoyl-2′-deoxytaxol N-benzoyltransferase
comp70594_c0_seq1	339521621	23.93	422	C-13 phenylpropanoid side chain CoA acyltransferase
comp70669_c0_seq1	28380187	34.95	432	taxa-4(20),11(12)-dien-5alpha-ol-O-acetyltransferase
comp68118_c0_seq1	28380187	28.44	450	taxa-4(20),11(12)-dien-5alpha-ol-O-acetyltransferase
comp53308_c0_seq1	386304662	38.04	163	taxadienol acetyl transferase, partial
comp119236_c0_seq1	53690152	45.98	87	taxadien-5-alpha-ol-O-acetyltransferase
comp68580_c0_seq1	53690152	30.06	173	taxadien-5-alpha-ol-O-acetyltransferase
comp37211_c0_seq1	53690152	32.39	142	taxadien-5-alpha-ol-O-acetyltransferase
comp83331_c0_seq1	53690152	28.84	215	taxadien-5-alpha-ol-O-acetyltransferase
comp64789_c0_seq1	53759170	42.6	446	taxadiene 5-alpha hydroxylase
comp57975_c0_seq1	386304485	50.32	155	taxadiene 5nalpha hydroxylase, partial
comp172528_c0_seq1	38201489	36.47	85	taxa-4(5),11(12)-diene synthase
comp193967_c0_seq1	15080743	46.03	63	taxadiene synthase
comp63152_c1_seq1	386304920	29.69	128	taxadiene synthase, partial
comp40035_c0_seq1	24266823	47.89	71	5-alpha-taxadienol-10-beta-hydroxylase
comp53405_c1_seq1	24266823	49.47	95	5-alpha-taxadienol-10-beta-hydroxylase
comp133851_c0_seq1	44903417	32.47	77	5-alpha-taxadienol-10-beta-hydroxylase
comp110423_c0_seq1	60459952	41.38	87	taxane 13-alpha-hydroxylase
comp69534_c0_seq1	60459952	33.79	441	taxane 13-alpha-hydroxylase
comp38773_c1_seq1	60459952	45.83	96	taxane 13-alpha-hydroxylase
comp36415_c0_seq1	60459952	34.19	427	taxane 13-alpha-hydroxylase
comp57975_c1_seq1	60459952	44.93	69	taxane 13-alpha-hydroxylase
comp104139_c0_seq1	60459952	42.17	83	taxane 13-alpha-hydroxylase
comp93979_c0_seq1	75297723	38.03	71	Taxane 14b-hydroxylase
comp61533_c1_seq1	380039801	33.57	143	taxane 14b-hydroxylase
comp143394_c0_seq1	380039801	29.17	120	taxane 14b-hydroxylase
comp74596_c0_seq1	380039801	29.51	122	taxane 14b-hydroxylase
comp84707_c0_seq1	380039801	35.22	230	taxane 14b-hydroxylase
comp36896_c0_seq1	67633430	30.84	467	taxoid 2-alpha-hydroxylase
comp192945_c0_seq1	238915468	43.75	64	taxoid 7-beta-hydroxylase
comp36946_c0_seq1	365776087	55	60	transcription factor WRKY
comp64330_c0_seq1	365776087	54.24	59	transcription factor WRKY
comp78449_c0_seq1	365776087	66.67	54	transcription factor WRKY
comp67132_c0_seq1	365776087	56.34	71	transcription factor WRKY
comp59687_c0_seq1	365776087	41.22	131	transcription factor WRKY
comp68275_c0_seq1	365776087	56.72	67	transcription factor WRKY
comp104123_c0_seq1	222355764	29.87	154	JAMYC
comp69212_c0_seq1	222355764	41.69	710	JAMYC
comp69212_c0_seq2	222355764	46.72	259	JAMYC
comp69212_c0_seq3	222355764	41.69	710	JAMYC
comp124731_c0_seq1	222355764	100	27	JAMYC
comp69971_c3_seq1	222355764	29.9	204	JAMYC
comp38183_c0_seq2	222355764	30	160	JAMYC
comp83061_c0_seq1	222355764	35.71	112	JAMYC

Protein GIs, instead of their accession numbers, are provided here for convenience in table layout. These can be queried at NCBI protein databases.

**Table 3:** **Proteins most homologous to genes involved in taxol synthesis in species outside *Taxus***

**Query Protein GI**	**Hit Protein GI**	**Identity (%)**	**Taxonomy**	**Hit Protein GI**	**Identity (%)**	**Taxonomy**
15080743	-	-	-	62511183	48.53	*Abies grandis*
38201489	-	-	-	62511183	48.66	*Abies grandis*
386304920	-	-	-	62511183	49.3	*Abies grandis*
24266823	56609042	97.99	*Ozonium* sp. BT2	75319884	43.97	*Picea sitchensis*
44903417	56609042	99.2	*Ozonium* sp. BT2	75319884	44.17	*Picea sitchensis*
53759170	56609042	66.38	*Ozonium* sp. BT2	75319884	44.44	*Picea sitchensis*
60459952	56609042	63.9	*Ozonium* sp. BT2	75319884	45.32	*Picea sitchensis*
67633430	56609042	56.34	*Ozonium* sp. BT2	75319884	40.89	*Picea sitchensis*
75297723	56609042	60.72	*Ozonium* sp. BT2	75319884	42.5	*Picea sitchensis*
238915468	56609042	56.2	*Ozonium* sp. BT2	75319884	40	*Picea sitchensis*
380039801	56609042	61.12	*Ozonium* sp. BT2	75319884	42.92	*Picea sitchensis*
386304485	56609042	66.22	*Ozonium* sp. BT2	75319884	47.11	*Picea sitchensis*
28380187	62461771	98.39	*fungal* sp. BT2^*^	148906373	43.98	*Picea sitchensis*
28558088	62461771	60.23	*fungal* sp. BT2	148906373	45.62	*Picea sitchensis*
53690152	62461771	60.83	*fungal* sp. BT2	148906373	44.25	*Picea sitchensis*
339521621	62461771	60	*fungal* sp. BT2	148906373	39.83	*Picea sitchensis*
386304662	62461771	98.23	*fungal* sp. BT2	148906373	44.07	*Picea sitchensis*
386304248	169135276	98.45	*Cladosporium cladosporioides*	148906373	44.64	*Picea sitchensis*
365776087	-	-	-	167859869	43.18	*Picea abies*
222355764	-	-	-	148906957	46.99	*Picea sitchensis*

* *Ozonium* sp. BT2 and *fungal* sp. BT2 are the same fungus species. [49,51].

Columns 2-4 show top hit protein information from fungi; columns 5-7 show top hit protein information from plants. Methodically, protein queries are blasted against NCBI nonredundant protein database and protein hits from the two designated sources with top sequence identity are recorded. Protein GIs, instead of their accession numbers, are provided here for convenience in table layout. These can be queried at NCBI protein databases. The reason for only three identified hit proteins from fungi is possibly due to the absence of genome data for taxol-producing fungi.

Overall, our study reported for the first time large-scale identification of genes involved in the terpenoid pathway in *Corylus*, which would facilitate understanding of taxol synthesis in angiosperms, although further experiments were required to clarify the roles of these genes in such processes. On the other hand, it should be noted that not all sequences of the genes related to taxol synthesis were revealed by the present transcriptome analyses because of difficulties in normalizing all cDNAs before sequencing when the level of leaf mRNA expression in the taxol synthesis pathway was very low. In addition, some taxadiene synthase genes might only be expressed in response to external stimuli, such as naturally occurring fungal infection or artificial chemical induction [47]. Such genes would not be detected by the present approach. Since the family of terpene synthases were highly diversified across plants [48], it would be interesting to investigate the reasons why taxol production was shared by these special gymnosperm and angiosperm plants. Horizontal gene transfer was a likely cause of such convergent evolutions via symbiotic organisms. For example, three genes from different taxol-producing fungi (two from *Ozonium* sp. BT2 and one from *Cladosporium cladosporioides*) isolated from the inner tree barks [49-51] had been shown high sequence identities (98.39%, 98.45% and 99.2%) to the corresponding taxol genes in yew species (Table 3). Undoubtedly, these unsolved questions merited further study, especially from genomic scanning and experimental tests.

## Conclusions

In the present study, the transcriptome of *C. mandshurica* was *de novo* assembled with Trinity and functionally annotated with Blast2go and KAAS. We found that highly differentiated genes between *C. mandshurica* and *C. avellana* correlated with local adaptation of the two species. In addition, a set of genes that might contribute to taxol production were identified and genetic mechanisms for taxol synthesis in distantly related plants were discussed. Thus, our study broadened the available transcriptome resources for *Corylus*, and provided meaningful information for researchers interested in taxol synthesis and high tolerance of *C. mandshurica* to fungal infection and cold stress.

## Methods

### Sequencing and assembly

Total RNA was extracted from leaves of *C. mandshurica* according to the CTAB protocol. The integrity of RNA was detected on an Agilent 2100 Bioanalyzer. The initial 20 μg of total RNA was purified using polydT conjugated beads to extract polyA-tagged mRNA, which was subsequently cleaved into ~200 bp fragments by treatment with divalent cations at 75°C. The first strand cDNA synthesis was carried out using reverse transcriptase (Invitrogen) with random hexamer primers, and the second strand using RNase H (Invitrogen) and DNA polymerase I (New England BioLabs). Sequencing was performed on an Illumina Genome Analyzer II.

After removal of adapter sequences, raw reads were filtered according to stringent criteria [52]. The clean reads generated were used for all subsequent analyses. Trinity was used to assemble the paired-end short reads into contigs.

### Functional annotation

The EST sequences were searched against the NCBI non redundant protein database using BLASTX with an E-value cutoff of 1e-5. The blast output in XML format was then annotated by Blast2go using default parameters. Kyoto Encyclopedia of Genes and Genomes (KEGG) was a universally acknowledged database for delineating networks of macromolecular interaction within cells. Pathway annotation was conducted using the KEGG Automatic Annotation Server (KAAS) web server and Single-directional Best Hit (SBH) method against representative sets for eukaryotes. GO enrichment was analyzed with WEGO.

### Ortholog identification and comparison

Bi-directional BLASTN searches were performed for the transcriptomes of *C. mandshurica* and *C. avellana*. The reciprocal best blast hits were considered as orthologs. Orthologs with sequence identity lower than 90% were discarded in further GO analyses in order to exclude distant homologs due to the incomplete and fragmentary nature of transcriptomes. Two types of sequence variations were studied in GO enrichment analyses. One type focuses on orthologs with relatively low sequence identity, which includes 10.4% of all orthologs with sequence identity less than 97%. The other focuses the presence of gaps in local alignments of orthologs as shown in BLASTN outputs. GO terms of all orthologs and these two types of orthologs were extracted from Blast2go outputs. GO enrichment analyses were carried out on WEGO server. GO terms with p-value of Pearson Chi-square test below 0.05 was considered statistically significant.

### The identification of genes involved in taxol synthesis

Genes related to taxol syntheses were identified by extensively parsing gene descriptions in the XML-formatted BLASTX output using key words of all the corresponding enzymes. The potential genes were further manually verified.

In order to compare these sequences with homologous genes in other species, we used the prototype genes responsible for taxol synthesis in yew as query sequences to search against the NCBI non redundant protein database using BLASTP. The top protein hits from fungi and plants were extracted.

### DATA Availability

Reads are deposited at NCBI SRA (SRR857924).

## Competing interests

The authors declare that they have no competing interests.

## Authors’ contributions

HM and JL analyzed the data and wrote the manuscript. ZL acquired the leaf sample. BL prepared the mRNA for sequencing. QQ provided helpful suggestion in data analysis. All authors read and approved the final manuscript.

## Supplementary Material

Additional file 1 **The assembled EST sequences of *C. mandshurica *in fasta format.** Click here for file

Additional file 2 GO terms for orthologs with sequence identity lower than 97. Click here for file

Additional file 3 GO terms for orthologs with INDEL. Click here for file

Additional file 4 GO terms for all orthologs. Click here for file

## References

[B1] Zhang Y Li F Tao R Li Z Liang Y An investigation of wild Corylus resource at Changbai Mountains J Jilin Agri Sci 2007 32 5 56 57

[B2] Liu H Exploring the utilization of Corylus Farm Prod Proc 2010 1 24 25

[B3] Huang M Selecting for excellent clones of Castanea henryi Fujian Agri Sci Technol 2012 12 35 40

[B4] Liu Y Zhang H Zhang W The new application of Corylus Spel Econ Anim Plant 1998 6 38

[B5] Plosker GL Hurst M Paclitaxel: a pharmacoeconomic review of its use in non-small cell lung cancer Pharmacoeconomics 2001 19 11 1111 1134 10.2165/00019053-200119110-00005 11735678

[B6] Kumar S Mahdi H Bryant C Shah JP Garg G Munkarah A Clinical trials and progress with paclitaxel in ovarian cancer Inter J Women’s Health 2010 2 411 427 21270965 10.2147/IJWH.S7012 PMC3024893

[B7] Gradishar WJ Taxanes for the treatment of metastatic breast cancer Bre Can: Basic Clin Res 2012 6 159 171 10.4137/BCBCR.S8205 PMC3486789 23133315

[B8] Wani MC Taylor HL Wall ME Coggon P McPhail AT Plant antitumor agents. VI. Isolation and structure of taxol, a novel antileukemic and antitumor agent from Taxus brevifolia J Am Chem Soc 1971 93 9 2325 2327 10.1021/ja00738a045 5553076

[B9] Vidensek N Lim P Campbell A Carlson C Taxol content in bark, wood, root, leaf, twig, and seedling from several Taxus species J Nat Prod 1990 53 6 1609 1610 10.1021/np50072a039 1982448

[B10] Service RF Hazel trees offer new source of cancer drug Science 2000 288 5463 27 28 10766629 10.1126/science.288.5463.27a

[B11] Bestoso F Ottaggio L Armirotti A Balbi A Damonte G Degan P Mazzei M Cavalli F Ledda B Miele M In vitro cell cultures obtained from different explants of Corylus avellana produce Taxol and taxanes BMC Biotechnol 2006 6 1 45 10.1186/1472-6750-6-45 17150090 PMC1702537

[B12] Hoffman A Shahidi F Paclitaxel and other taxanes in hazelnut J Funct Foods 2009 1 1 33 37 10.1016/j.jff.2008.09.004

[B13] Ottaggio L Bestoso F Armirotti A Balbi A Damonte G Mazzei M Sancandi M Miele M Taxanes from Shells and Leaves of Corylus avellana J Nat Prod 2007 71 1 58 60 18163585 10.1021/np0704046

[B14] Luo F Fei X Tang F Li X Simultaneous determination of Paclitaxel in hazelnut by HPLC-MS/MS For Res 2011 24 6 779 783

[B15] Miele M Mumot A Zappa A Romano P Ottaggio L Hazel and other sources of paclitaxel and related compounds Phytochem Rev 2012 11 2–3 211 225

[B16] Liu X-K Liu J-J New source for L-iditol and taxanes Nat Preced 2008 http://precedings.nature.com/documents/1502/version/1

[B17] Bemani E Ghanati F Rezaei A Jamshidi M Effect of phenylalanine on taxol production and antioxidant activity of extracts of suspension-cultured hazel (Corylus avellana L.) cells J Nat Med 2013 67 3 446 451 10.1007/s11418-012-0696-1 22847380

[B18] Zhao D Su S Ni B Wang W Meng X Liu W Germplasm resources investigation and utilization prospects of hazel in Small Xing’an Ridge region Chin Agric Sci Bull 2012 28 28 87 94

[B19] Coyne CJ Mehlenbacher SA Smith DC Sources of resistance to Eastern Filbert Blight in hazelnut J Am Soc Hortic Sci 1998 123 2 253 257

[B20] Molnar TJ Capik J Zhao S Zhang N First report of Eastern Filbert Blight on Corylus avellana 'Gasaway’ and 'VR20-11’ caused by Anisogramma anomala in New Jersey Plant Dis 2010 94 10 1265 1265 10.1094/PDIS-06-10-0445 30743588

[B21] Liang W An investigation of wild Corylus resources in China Journal of Liaoning Forestry Science and Technology 1989 1 45 52

[B22] Peng L Wang M Liang W Xie M Li D **A study on cold resistance for filbert genus (Corylus L.) plants** J Jilin Fores Univ 1994 3 166 170

[B23] Ni B Ni W Xu X Wang X Hazel breeding research Forest By-Product and Speciality in China 2010 106 3 29 31

[B24] X-j Z F-x D R-q Z G-x W M-p Y L-s L Research on the compatibility of five Corylus species J Cen South Univ Fores Technol 2009 29 4 26 30

[B25] Erdogan V Mehlenbacher SA Interspecific hybridization in hazelnut (Corylus) J Am Soc Hortic Sci 2000 125 4 489 497

[B26] Liang W Xie M Dong D Jiang Z The breeding research for new Corylus cultivar China Fruits 2000 2 4 6

[B27] Cheng L Huang W Zhou Z Liu J Wang Y Su S Zhai M Genetic diversity of six Corylus species in China detected with microsatellite isolated from Corylus avellana Scientia Silvae Sinicae 2009 45 2 22 26

[B28] Li X Li X Wang Z Xue C Limin Z Guo Y Study on phylogenetic analysis of Corylus germplasm resouces with SSR molecular markers for Corylus avellana J Northeast Agric Univ 2011 42 4 129 136

[B29] Rowley ER Fox SE Bryant DW Sullivan CM Priest HD Givan SA Mehlenbacher SA Mockler TC Assembly and characterization of the European hazelnut 'Jefferson’ transcriptome Crop Sci 2012 52 6 2679 2686 10.2135/cropsci2012.02.0065

[B30] Grabherr MG Haas BJ Yassour M Levin JZ Thompson DA Amit I Adiconis X Fan L Raychowdhury R Zeng Q Full-length transcriptome assembly from RNA-Seq data without a reference genome Nat Biotechnol 2011 29 7 644 652 10.1038/nbt.1883 21572440 PMC3571712

[B31] Min XJ Butler G Storms R Tsang A OrfPredictor: predicting protein-coding regions in EST-derived sequences Nucleic Acids Res 2005 33 suppl 2 W677 W680 15980561 10.1093/nar/gki394 PMC1160155

[B32] Wang N Thomson M Bodles WJA Crawford RMM Hunt HV Featherstone AW Pellicer J Buggs RJA Genome sequence of dwarf birch (Betula nana) and cross-species RAD markers Mol Ecol 2013 22 11 3098 3111 10.1111/mec.12131 23167599

[B33] Kent WJ BLAT—The BLAST-Like Alignment Tool Genome Res 2002 12 4 656 664 11932250 10.1101/gr.229202 PMC187518

[B34] Altschul SF Madden TL Schäffer AA Zhang J Zhang Z Miller W Lipman DJ Gapped BLAST and PSI-BLAST: a new generation of protein database search programs Nucleic Acids Res 1997 25 17 3389 3402 10.1093/nar/25.17.3389 9254694 PMC146917

[B35] Conesa A Götz S García-Gómez JM Terol J Talón M Robles M Blast2GO: a universal tool for annotation, visualization and analysis in functional genomics research Bioinformatics 2005 21 18 3674 3676 10.1093/bioinformatics/bti610 16081474

[B36] Moriya Y Itoh M Okuda S Yoshizawa AC Kanehisa M KAAS: an automatic genome annotation and pathway reconstruction server Nucleic Acids Res 2007 35 suppl 2 W182 W185 17526522 10.1093/nar/gkm321 PMC1933193

[B37] Srivastava A Cai L Mrázek J Malmberg RL Mutational patterns in RNA secondary structure evolution examined in three RNA families PLoS ONE 2011 6 6 e20484 10.1371/journal.pone.0020484 21698102 PMC3117835

[B38] Pelletier J Sonenberg N Insertion mutagenesis to increase secondary structure within the 5’ noncoding region of a eukaryotic mRNA reduces translational efficiency Cell 1985 40 3 515 526 10.1016/0092-8674(85)90200-4 2982496

[B39] Brown PH Tiley LS Cullen BR Effect of RNA secondary structure on polyadenylation site selection Genes Dev 1991 5 7 1277 1284 10.1101/gad.5.7.1277 1712333

[B40] McManus CJ Graveley BR RNA structure and the mechanisms of alternative splicing Curr Opin Genet Dev 2011 21 4 373 379 10.1016/j.gde.2011.04.001 21530232 PMC3149766

[B41] Mauger DM Siegfried NA Weeks KM The genetic code as expressed through relationships between mRNA structure and protein function FEBS Lett 2013 587 8 1180 1188 10.1016/j.febslet.2013.03.002 23499436 PMC4269304

[B42] Amini F Ismail E 3′-UTR variations and G6PD deficiency J Hum Genet 2013 58 4 189 194 10.1038/jhg.2012.155 23389243

[B43] Ye J Fang L Zheng H Zhang Y Chen J Zhang Z Wang J Li S Li R Bolund L WEGO: a web tool for plotting GO annotations Nucleic Acids Res 2006 34 suppl 2 W293 W297 16845012 10.1093/nar/gkl031 PMC1538768

[B44] Xiong Z Yang Y Zhao N Wang Y Diversity of endophytic fungi and screening of fungal paclitaxel producer from Anglojap yew. Taxus x media BMC Microbiol 2013 13 1 71 10.1186/1471-2180-13-71 23537181 PMC3618195

[B45] Li S Zhang P Zhang M Fu C Yu L Functional analysis of a WRKY transcription factor involved in transcriptional activation of the DBAT gene in Taxus chinensis Plant Biol 2013 15 1 19 26 10.1111/j.1438-8677.2012.00611.x 22686366

[B46] Nims E Vongpaseuth K Roberts SC Walker EL WITHDRAWN: TcJAMYC: A bHLH transcription factor that activates paclitaxel biosynthetic pathway genes in yew (This manuscript was accepted by the Journal of Biological Chemistry, but following acceptance discrepancies in some of the sequences used in the work were discovered. The manuscript was withdrawn, and additional work has been conducted. Submission of a new manuscript is anticipated.) J Biol Chem 2009 http://www.jbc.org/content/early/2009/10/01/jbc.M109.026195

[B47] Sun G Yang Y Xie F Wen J-F Wu J Wilson IW Tang Q Liu H Qiu D Deep sequencing reveals transcriptome re-programming of Taxus × media cells to the elicitation with methyl jasmonate PLoS ONE 2013 8 4 e62865 10.1371/journal.pone.0062865 23646152 PMC3639896

[B48] Chen F Tholl D Bohlmann J Pichersky E The family of terpene synthases in plants: a mid-size family of genes for specialized metabolism that is highly diversified throughout the kingdom Plant J 2011 66 1 212 229 10.1111/j.1365-313X.2011.04520.x 21443633

[B49] Guo BH Wang YC Hu H Miao ZQ Tang KX An endophytic Taxol-producing fungus BT2 isolated from Taxus chinensis var. maire Afr J Biotechnol 2006 5 10 875 877

[B50] Zhang P Zhou P-P Yu L-J An endophytic taxol-producing fungus from Taxus media, Cladosporium cladosporioides MD2 Curr Microbiol 2009 59 3 227 232 10.1007/s00284-008-9270-1 19484305

[B51] Wei Y Zhou X Liu L Lu J Wang Z Yu G Hu L Lin J Sun X Tang K An efficient transformation system of taxol-producing endophytic fungus EFY-21 (Ozonium sp.) Afr J Biotechnol 2010 9 12 1726 1733

[B52] Qiu Q Ma T Hu Q Liu B Wu Y Zhou H Wang Q Wang J Liu J Genome-scale transcriptome analysis of the desert poplar. Populus euphratica Tree Physiol 2011 31 4 452 461 10.1093/treephys/tpr015 21427158

